# Some Candidate Drugs for Pharmacotherapy of Alzheimer’s Disease

**DOI:** 10.3390/ph14050458

**Published:** 2021-05-13

**Authors:** Barbara Miziak, Barbara Błaszczyk, Stanisław J. Czuczwar

**Affiliations:** 1 Department of Pathophysiology, Medical University of Lublin, 20-090 Lublin, Poland; barbaramiziak@umlub.pl; 2 Faculty of Health Sciences, High School of Economics, Law and Medical Sciences, 25-734 Kielce, Poland; barbarablaszczyk@op.pl

**Keywords:** Alzheimer’s disease, antidepressants, antidiabetics, bexarotene, cognitive, curcumin, myricetin, resveratrol

## Abstract

Alzheimer’s disease (AD; progressive neurodegenerative disorder) is associated with cognitive and functional impairment with accompanying neuropsychiatric symptoms. The available pharmacological treatment is of a symptomatic nature and, as such, it does not modify the cause of AD. The currently used drugs to enhance cognition include an N-methyl-d-aspartate receptor antagonist (memantine) and cholinesterase inhibitors. The PUBMED, Medical Subject Heading and Clinical Trials databases were used for searching relevant data. Novel treatments are focused on already approved drugs for other conditions and also searching for innovative drugs encompassing investigational compounds. Among the approved drugs, we investigated, are intranasal insulin (and other antidiabetic drugs: liraglitude, pioglitazone and metformin), bexarotene (an anti-cancer drug and a retinoid X receptor agonist) or antidepressant drugs (citalopram, escitalopram, sertraline, mirtazapine). The latter, especially when combined with antipsychotics (for instance quetiapine or risperidone), were shown to reduce neuropsychiatric symptoms in AD patients. The former enhanced cognition. Procognitive effects may be also expected with dietary antioxidative and anti-inflammatory supplements—curcumin, myricetin, and resveratrol. Considering a close relationship between brain ischemia and AD, they may also reduce post-brain ischemia neurodegeneration. An investigational compound, CN-105 (a lipoprotein E agonist), has a very good profile in AD preclinical studies, and its clinical trial for postoperative dementia is starting soon.

## 1. Introduction

Alzheimer’s disease (AD) is a neurodegenerative, debilitating, and fatal disease characterized by progressive cognitive impairment, behavioral disorders, and loss of function in daily life. AD is the most common cause of dementia, accounting for 50–70% of dementia cases worldwide [1]. The 2018 World Alzheimer’s Disease Report shows that 50 million people worldwide have dementia [2].

AD has several neuropathological hallmarks, including the deposition of β-amyloid (Aβ) peptides in the extracellular matrix between neurons (known as amyloid plaques), the intracellular formation of neurofibrillary tangles arising from the accumulation of hyperphosphorylated tau protein in neurons, neuronal loss, neuroinflammation, and oxidative stress [3,4,5]. It has been reported that amyloid accumulation in brain tissue can be observed up to 10–20 years before the onset of clinical symptoms [6]. Risk factors for AD include age, family history, apolipoprotein E_4 (apoE) genotype, diabetes, hypertension, obesity, hypercholesterolemia, traumatic brain injury, and low education level [7]. Mutations in the genes presenilin 1, presenilin 2, and amyloid precursor protein (*APP*) are associated with early-onset autosomal-dominant AD [7]. At present, only symptomatic but not disease-modifying drug treatments are available.

## 2. Methods

Candidate drugs for pharmacotherapy of AD were searched for in English language articles with the use of three databases: PUBMED, Clinical Subject Heading and Clinical Trials. The search areas included the following keywords: AD and novel treatment, Aβ and modifying drugs, and AD and clinical trials. Particularly important references were also extracted from publications found in databases.

## 3. Current Pharmacological Treatment

Despite the growing population of patients with AD, only five treatment options (Table 1) are currently approved to treat the cognitive symptoms of AD in the United States, the most recent of which, memantine (Namenda, 20 mg per day), as a N-methyl-d-aspartate receptor antagonist, was approved more than a decade ago [7,8]. The addition of memantine should be considered for the treatment of cognitive and functional symptoms in patients with moderate to severe AD or mixed dementia who are already receiving a cholinesterase inhibitor [3].

Four of the five standard-of-care treatments are also licensed in the European Union; these include three cholinesterase inhibitors and one N-methyl-d-aspartate receptor antagonist (memantine) [7]. Cholinesterase inhibitors, including donepezil (Aricept, 5 to 10 mg per day), galantamine (Razadyne, at least 16 mg per day), or rivastigmine (Exelon, 6 to 12 mg per day orally or 9.5 mg per day transdermally), should be considered for treatment of cognitive and functional decline in patients with mild to moderate AD (evidence rating A) [3]. When switching from oral to transdermal administration, if the total daily dosage of galantamine is less than 6 mg, it is recommended to use a 4.6 mg patch. If the total daily dosage is 6 to 12 mg, a 9.5 mg patch may be used. On the other hand, the American Geriatrics Society does not recommended prescribing cholinesterase inhibitors for dementia without periodic assessment for perceived cognitive benefits and adverse gastrointestinal effects [3].

In 2014, a fifth treatment option consisting of a fixed-dose combination with donepezil and memantine was approved for the treatment of patients with moderate to severe AD dementia who are on stable donepezil therapy [7]. Most therapeutic agents under development over the past 15 years have failed; AD is among the least well-served therapeutic areas for drug treatments. Nearly all trials conducted to date have been monotherapy trials comparing an active agent with placebo with or without a background standard-of-care agent, such as cholinesterase inhibitors or memantine [7].

There has also been a proposal for a memantine/donepezil combination with vitamin E. The addition of vitamin E (2000 IU per day) should be considered for the treatment of mild to moderate AD in patients who are already receiving a cholinesterase inhibitor (B) [3].

In addition to pharmacological therapy, patients are also recommended non-pharmacological therapy [3]. A structured physical exercise program should be recommended for patients with AD of any severity (A) [3]. Cognitive stimulation programs should be recommended for patients with mild to moderate cognitive impairment (B) [3].

In addition, there are specific recommendations for patients with AD, including:

- Enjoyable leisure activities (per patient preference; mild cognitive impairment, mild to moderate dementia)—decreased neuropsychiatric symptoms and functional capacity, slowing memory loss.
- Mental stimulation programs (e.g., puzzles, word games, past/reminiscence therapy, indoor gardening, baking; mild to moderate dementia)—evidence for improved cognition and self-reported quality of life and well-being; no effect on functional status, mood, or behavior.
- Occupational therapy training in coping strategies and cognitive aides (mild to moderate dementia)—improved cognition.
- Structured physical exercise programs (mild to severe AD)—improved physical function, reduced neuropsychiatric symptoms (including depression), slower rate of functional decline, no improvement in cognition.

Although continuous dose optimization is an option for treating all stages of AD, certain patients may fail to achieve sustained clinical benefits from cholinesterase inhibitors, sometimes resulting in discontinuation of the treatment [4]. In these patients, switching between cholinesterase inhibitors is a reasonable therapeutic option because it is crucial to not give up on treatment after the first therapy has failed owing to a lack of clinical benefit [4]. In a multicenter, 2-year prospective study, the incidence of switching between cholinesterase inhibitors was 9.2 per 100 person-years among 611 patients treated at baseline [4]. The use of a ketogenic diet in various diseases has been growing recently [9]. The best results relating to ketogenic diet treatment are expected in early presymptomatic stages of AD [9]. Due to the high prevalence of AD, and its high economic burden to society, there is significant interest in developing new approaches to treat AD [10].

Despite drug trials being conducted in many centers around the world, satisfactory results have still not been obtained. Different theories about the pathomechanisms in AD are regularly put forward. One such theory which was initially positively promising was the research on the beta-site amyloid precursor protein cleaving enzyme 1 (BACE1) inhibitor. BACE1 is involved in the formation of monomeric forms of amyloid-β (Aβ), including Aβ42. Furthermore, it has been shown that the concentration and activity of BACE1 is increased in both the brains and body fluids of AD patients, so the idea of research into BACE1 inhibitors seemed to be a very good idea. Several clinical trials of BACE1 inhibitors have even been initiated, but to date all have been discontinued for futility or safety reasons [11].

As already mentioned, AD is associated with deposition of amyloid-β protein (Aβ) as β-amyloid plaques, but the known mutation that contributes to peptides generation leads to early-onset familial AD. The above mutation has not been observed in other AD patients. More recent studies extend the concept of pathomechanisms also to tau neurofibrillary degeneration, inflammatory reactions of microglia and astrocytes, mitochondrial dysfunction as well as autophagy and mitophagy or blood–brain barrier permeability abnormalities [12,13,14]. Other studies also point to the involvement of gut microflora, carbonate disturbances, including cerebral insulin resistance [6,15,16].

## 4. APOE and Alzheimer’s Disease

APOE is a gene that encodes apoE, having three different isoforms (E2, E3 and E4). The E3 allele is the most common form in the population and accounts for approximately 80% of allelic variation, while the frequency of E2 and E4 is less than 7% and 15%, respectively. ApoE E4 is a recognized risk factor for AD, in contrast to apoE E2, which has a protective effect and significantly reduces the risk of developing late-onset AD [12,17]. In vivo studies in genetically modified rodent models (have a human APOE knock-in background) have attempted approaches to improve phenotypes characteristic of AD patients. Unfortunately, although a lot of research in this direction has started, much less continues [12].

One of the treatment suggestions was to increase the concentration and lipidation of APOE in the brain. It was observed that deletion of the ABCA1 gene induced poor apoE lipidation and increased Aβ plaque burden. On the other hand, when ABCA1 is overexpressed, a reduction in Aβ accumulation is noted. Stimulation of the retinoid X receptor induces the expression of ABCA1 as well as ABCG1 [18,19].

Bexarotene, a drug used in cancer therapy, is a retinoid X receptor agonist [20]. Recently, the US Food and Drug Administration approved it as a drug for use in patients with cutaneous T-cell lymphoma [12]. More recent published data also revealed the effect of bexarotene on an intensive reduction in Aβ plaques and improvement of cognitive function in three genetically modified mouse models of AD (APPswe/PSEN1dE9—at a dose of 100 mg/kg for 3, 7, 14 or 90 days; APP/PS1-21—at a dose of 100 mg/kg for 20 days; and Tg2576—at a dose of 100 mg/kg for 3 or 7 days). In all three models, the drug was administered orally. The results point to reduced soluble or insoluble Aβ and Aβ plaques, improved memory and increased HDL levels, most likely related to increased ATP-binding cassette transporter (a product of ABCA1 gene), ATP-binding cassette sub-family G member 1 (a protein encoded by the ABCG1 gene) expression and apoE lipidation [19]. Similar results were obtained with a bexarotene dose of 100 mg/kg for 30 days in very old triple transgenic AD mice (3xTg-AD mice). The authors demonstrated improved cognitive function, as well as baseline synaptic transmission and synaptic plasticity, reduced astrogliosis and reactive microglia in both the cortex and hippocampus. In addition, increased expression of APOE was found, but limited to CA1 hippocampal subfield [21].

In contrast, Fitz et al. [22], treating APP/PS1ΔE9 mice, obtained similar effects on the reduction in memory deficits and significant decrease in interstitial fluid Aβ, but no effect on amyloid deposition was found. Similarly, Veeraraghavalu et al. [23] showed only a reduction in soluble Aβ40 levels in mice, but no effect of the drug on the plaque burden that exhibits Aβ amyloidosis.

Clinical trials evaluating the effects of bexarotene have been conducted. Ghosal et al. [24] conducted a randomized double-blind, placebo-controlled trial (proof-of-mechanism trial, phase Ib) involving healthy, young (12 participants, mean age approximately 30–32 years) subjects with the APOE ε3/ε3 genotype. Volunteers received orally for 3 days a placebo or 225 mg of bexarotene twice daily (at 9 a.m. and 7 p.m.). After analysis of the results, the drug was found in plasma at concentrations of 1 to 2 μM, with very low concentrations in cerebrospinal fluid (CSF) (in nM). Furthermore, bexarotene increased apoE concentrations by 25%, and no changes in Aβ peptide metabolism were demonstrated [24]. Among the side effects, increases in triglycerides (three people—over 200 mg/dl) and total cholesterol (one person—over 200 mg/kg) were observed. In addition, thyroid dysfunction and increases in liver enzymes were observed (two people each), as well as isolated cases of nausea, headache or rash. All adverse effects were judged to be non-significant, which spontaneously resolved by the end of the study [24].

Cummings et al. [25] conducted a double-blind, placebo-controlled, proof-of-concept trial in which patients with moderate AD were administered 300 mg of bexarotene or placebo for 4 weeks. The results show no change in amyloid burden in apoE4 carriers, while in apoE4 noncarriers, a significant association was observed between increased serum Aβ1-42 and reductions in brain amyloid. Among side effects, a large increase in triglycerides (more than 200 mg/dL) in most patients and an increase in total cholesterol of more than 300 mg/dL in half of the subjects, representing a high risk of cardiovascular disease, as well as isolated cases of symptoms such as delusions, dizziness, dry cough, and toe blister or diverticulitis, were demonstrated [25].

Another candidate for the treatment of patients with AD is ADCS-6253, a peptide, a C-terminal apoE derivative, which directly activates ABCA1 expression, as demonstrated in vitro. In vivo studies have been performed with young genetically modified mouse models (APOE3 knock-in and APOE4 knock-in) [25]. In APOE4 knock-in mice, an increase in apoE4 lipidation AND ABCA1 expression and “*a reversal of the apoE4-driven Aβ42 accumulation and tau hyperphosphorylation in hippocampal neurons, as well as of the synaptic impairments and cognitive deficits*” were observed. The above changes were not observed in APOE3 knock-in mice [26].

The available data suggest that apoE4 facilitates Aβ deposition, but the exact mechanisms are not known. Hori et al. [27] conducted a study in which injected Aβ protofibrils induced Aβ deposition in the brain of APP transgenic mice, demonstrating a correlation of Aβ protofibrils and Aβ accumulation. In the next step, Aβ protofibrils were injected with apoE3, resulting in a reduction in Aβ deposition. On the other hand, when combined with apoE4, no such effect was observed [27]. In vitro studies indicate that conversion of Aβ protofibrils to fibrils occurs faster in correlation with apoE4 and are more stable than in combination with apoE2 or apoE3 [27]. This explains why 2.7 times lower levels of Aβ oligomers are found in the brains of apoE3 AD patients [28]. An interesting idea then is to impede the interaction of apoE4 and Aβ [12,29]. Studies have been conducted in mouse models of AD disease in this direction using monoclonal anti-apoE antibodies as well as small molecules that act as Aβ mimetics [27,28].

Liao et al. [30] showed that administration of HJ6.3, a monoclonal antibody against apoE (at a dose of 10 mg/kg/week for 21 weeks) to APPswe/PS1ΔE9 (APP/PS1) mice, decreased soluble and insoluble Aβ and microglia, slightly decreased brain Aβ plaques and increased plasma Aβ. In addition, a slight improvement in spatial learning performance was observed, but no effect of the antibody on total blood cholesterol or cerebral amyloid angiopathy was noted. To evaluate the mechanisms of immunotherapy, HJ6.3 antibodies were applied to the surface of the cerebral cortex. The results of imaging studies (over a period of 2 weeks) show that the antibody prevented the formation of new Aβ deposits, reduced the growth of existing deposits, and even the occasional removal of amyloid plaques was observed [30]. Another antibody was also tested—“*anti-human apoE antibody, anti-human apoE 4 (HAE-4), that specifically recognizes human apoE4 and apoE3 and preferentially binds nonlipidated, aggregated apoE over the lipidated apoE found in circulation*” in APPPS1-21 x APOE4 knock-in mice. HAE-4 (administered centrally or by peripheral injection) reduced the accumulation of Aβ in the brain [31].

Krishnamurthy et al. investigated the effect (over a period of 40 days) of an apoE mimetic CN-105 in APP/PS1/APOETR mice [32]. Analysis of the results indicated that the mimetic reduced soluble Aβ and Aβ plaques as well as improved memory. In addition, the authors provided evidence that better effects were obtained when the mimetic was applied at an early stage of the disease [32]. This compound (at doses of 0.1, 0.5, or 1 mg/kg) will also be tested for its effects in postoperative dementia in a randomized, double-blind, placebo-controlled trial (phase 2) in adults (aged ≥ 60). The study is at the patient recruitment stage (NCT03802396).

Since apoE is undeniably responsible for the accumulation of Aβ in brains, there has been a proposal to reduce apoE levels. In vivo studies have shown that apoE genetic deletion or haploinsufficiency in fact reduced Aβ accumulation [33] and tau-induced neurodegeneration in tauopathy [34]. Another way to decrease Aβ is to increase the expression of apoE receptors, in that increased expression of low-density lipoprotein receptor (LDLR) results in decreased deposition of amyloid plaques in the brain. This is because LDLR increases the transport of Aβ from the brain into the blood [35]. Another option to lower brain apoE levels is to silence APOE expression with specific antisense oligonucleotides. Studies were performed on mouse models of AD, including APPPS1-21 × APOE3 knock-in mouse and APPPS1-21 × APOE4 knock-in mice. The experiments showed a reduction in soluble apoE concentrations by half with anti-APOE antisense oligonucleotides by approximately 50%. Such a change contributed to a reduction in Aβ concentrations (both soluble and insoluble Aβ forms), Aβ plaques and dystrophic neurites. Similar effects were obtained when the compound was administered intracerebrally at birth as well as early in the course of the disease (approximately 6-week-old mice) [36].

## 5. Insulin and Other Antidiabetics as a Potential Therapy for Alzheimer’s Disease

Patients with Alzheimer’s disease often have glucose metabolism abnormalities leading to type 2 diabetes [37].

Thus, another attempt to treat AD is the use of intranasal insulin treatment. Some studies have shown that acute and prolonged intranasal insulin administration alleviates AD neuropathology. Significant improvements were found in memory performance and synaptic plasticity, while increases in regional glucose uptake were also observed [38].

Previous studies have shown a close association between diabetes, insulin resistance and mild cognitive and memory impairment. Additionally, it is noted that patients with impaired brain energy utilization have a significantly higher risk of AD. This is because diabetes attenuates the amyloid precursor protein metabolism, which increases the deposition of β-amyloid (as a consequence of abnormal removal of Aβ plaques), increases tau protein phosphorylation and increases glycogen synthase kinase 3β levels [15,39]. In addition, sustained chronically high peripheral insulin levels interfere with insulin transport across the blood–brain barrier. This in turn alters insulin signaling in the brain, resulting in anti-apoptotic effects [40]

Additionally, in patients with high insulin levels, increased levels of inflammatory factors, oxidative stress and mitochondrial dysfunction were observed. In addition, neuroimaging studies showed damage to cerebral vessels [15,39,40]. The existence of common pathways in the pathomechanism is also important evidence for the coexistence of the relationship between diabetes and AD, including enzymatic degradation of Aβ, forehead box protein O1 (FOXO) signaling or insulin signaling [41].

Vandal et al. [16] performed experiments on a genetic mouse model of 3xTg-AD. This model (animal model of type 2 diabetes) is characterized by hyperinsulinemia and hyperglycemia (hippocampus in particular). In addition, animals were found to have low CSF-insulin levels and low insulin-mediated glucose disposal. The use of a high-fat diet in this mouse model resulted in “*cerebral expression of human AD transgenes led to peripheral glucose intolerance, associated with pancreatic human Aβ accumulation*”. In addition, increased glucose intolerance, elevated brain soluble Aβ, as well as memory impairment in rodents were observed. In a further step, it was shown that a single insulin (3.8 units/kg of human insulin) injection reversed the impairment caused by a high-fat diet, which the authors explained is most likely due to changes in Aβ production and/or clearance [16].

Other studies have shown that even systemic administration of exogenous insulin can contribute to the onset of memory impairment and neuronal dysfunction. On the other hand, there are studies indicating that the induced hyperinsulinemia to uphold the euglycemia can improve memory, which has been shown in the healthy brain as well as in AD. In animal studies, it has also been demonstrated that an acute increase in blood glucose levels can improve memory, which is related, among other things, to an increase in cholinergic activity. It should be remembered, however, that in the case of chronic hyperglycemia, as occurs in untreated/poorly treated diabetes, the effects are the opposite and cause cognitive impairment, particularly in older people [42].

The available literature shows that the way in which insulin is delivered to the body is also important for its action in AD patients [43]. Weinstein et al. [44] reported that peripheral insulin use increases the risk of dementia by 50%. These authors attributed this effect to the hypoglycemic action of insulin and the brain dysfunction associated with hypoglycemia [44].

On the other hand, other studies indicated that the use of intranasal insulin reduces cognitive impairment and the risk of developing dementia, as shown by Maimaiti et al. [45]. The authors evaluated the effects of insulin administration in two forms: short-acting insulin lispro (Humalog—the dose received 1–3 h prior to memory testing) or long-acting insulin detemir (Levemir—for 8–11 days) on cognitive function in ageing F344 rats. Additionally, insulin’s effects on the Ca^2+^-dependent hippocampal after hyperpolarization (AHP), a neurophysiological marker that increases in aging animals with memory impairment, were investigated. The results clearly indicate that the use of low-dose insulin (0.0715 IU/day/rat) in both treatment regimens reduced AHP and overall, significant improvements in memory tasks were evident [45].

It has also been reported that the effect of intranasal insulin may depend not only on the treatment regimen (acute vs. subchronic), but even on the sex and age of the patient and the APOE genotype [46].

Reger et al. [47] studied the effect of intranasal insulin in patients with different APOE genotypes—with (epsilon4+) and without (epsilon4-) the APOE- epsilon4 allele. Patients (memory-impaired adults with AD or amnestic mild cognitive impairment vs. control group) were given five intranasal treatment conditions consisting of insulin (10, 20, 40, or 60 IU) or placebo. Behavioral tests were performed after 15 min and blood was drawn after 45 min. The results show improvement in “*verbal memory in memory-impaired epsilon4- adults, with performance generally peaking at 20 IU. In contrast, memory-impaired epsilon4+ subjects demonstrated a relative decline in verbal memory*”. Changes in plasma amyloid-beta levels were also observed for memory-impaired subjects and normal controls, effects that again differed by APOE genotype. Intranasal insulin administration had no effect on insulin levels and plasma glucose [47].

Another research group has shown that acute intranasal insulin (40 I.U) improves odor-induced spatial memory. The study was conducted in a double-blind, placebo-controlled, counterbalanced within-subject design in young men. [48].

Data are also available that state no effect of such therapy. In recent months, the first multicenter, randomized, double-blind (phase 2/3) clinical trial evaluating the feasibility, safety and efficacy of intranasal insulin in the treatment of patients (ages 55 to 85) with mild cognitive impairment and dementia in AD was released. Participants received 40 IU of insulin (administered with 2 intranasal delivery devices) or placebo daily for 12 months followed by a 6-month open-label extension phase. However, the results were not as expected. The first device used for 49 patients to administer insulin had inconsistent reliability, so only the second device was used for the remaining 240 participants. In this clinical trial, no cognitive and functional benefits were observed with intranasal insulin treatment over. There were also no changes in CSF biomarker levels. The only difference shown between the study group and placebo was a slight reduction in hippocampal volume, but this change is of unclear clinical significance [49].

Another research group carried out a single-center, randomized, double-blind, placebo-controlled study to evaluate the efficacy of intranasal glulisine—rapid-acting insulin (at a dose of 20 IU twice daily). The study group were subjects with amnestic mild cognitive impairment or mild probable AD. The results show, as in previous studies, that glulisine did not affect plasma insulin or glucose levels. There was also no effect of the drug on patients’ cognitive function and memory [50].

Summing up intranasal insuline, its clinical use seems safe but, as suggested by Hallschmid [51], more data from broad clinical trials are required in order to compare responses from female and male patients and optimize delivery devices.

Batista et al. [52] investigated the effects of liraglutide, a glucagon-like peptide-1 analog, in different experimental models of AD (hippocampal cell cultures, mouse model and the infusion of amyloid-β oligomers (AβOs) into the lateral cerebral ventricle of non-human primates (NHPs)). In the mouse model, memory impairment was induced by AβOs (at a dose of 10 pmol) injected into the lateral ventricle. AβOs “*are small, diffusible aggregates of the Aβ peptide that accumulate in AD brains*”. The presence of AβOs causes changes similar to those seen in AD, including neuronal tau hyperphosphorylation, increased oxidative stress, and inhibition of synaptic plasticity, and this gives a clinical picture in the form of memory and cognitive dysfunction. Furthermore, impaired insulin signaling induces re-entry into the neuronal cell cycle, resulting in neuronal death [52,53,54,55]. In turn, in the case of NHPs, AβOs induce changes such as brain inflammation, loss of synapses, as well as tau hyperphosphorylation and tangle formation [52,56]. The authors obtained favorable results after liraglutide was administered (i.p) at a dose of 25 nmol/kg for 7 days. In vitro studies (hippocampal neuronal cultures) have shown that liraglutide has neuroprotective effects through activation of the protein kinase A (PKA) signaling pathway. Treatment with liraglutide in a third research model (NHPs) reduced AD-related insulin receptor, synaptic and tau pathologies in specific brain regions [52]. Another drug being investigated is metformin. However, studies to date are quite controversial. Some of the available data indicate a protective effect of the drug (especially with long-term therapy) by slowing the rate of cognitive decline [57]. Other studies show no association between metformin use and cognitive impairment, similar to that of sulfonylurea [44,58]. Another drug from this group, pioglitazone, was reported in three studies to improve cognitive performance in diabetic patients with AD; however, two studies with the use of this drug provided negative data [59].

## 6. Intestinal Microbiota and Alzheimer’s Disease

In addition to the already mentioned factors involved in the development of AD, there has been recent information about the role of the intestinal microbiota in the pathogenesis of the disease. This concept is related to the existence of the so-called “gut-brain-microbiota axis”. Therefore, there are speculations about the possibility of modulating this axis for prophylactic and perhaps even therapeutic purposes for AD disease [6,60]. The correlation between the gut and the brain may be based on, among other things, the interaction via specific and non-specific chemical compounds produced by the gut flora that cross the blood–brain barrier (BBB). Such compounds include glutamate, monoamines, methionine, serotonin, dopamine and histamine, as well as amino acid metabolites, homocysteine, GABA and tryptophan, and these may, once they reach central neurons, affect their activity [61,62]. In addition, the available data indicate that the gut microflora may also influence the NMDA receptors, brain-derived neurotrophic factor and serotonin receptors, which in turn regulate synaptic plasticity [63]. On the other hand, the nervous system via neurotransmitters can influence the functioning of the gut bacteria [64,65,66]. In addition, the intestinal mucosa as well as the BBB allow the passage of hormones and cytokines, which can affect both the brain and the gut. In summary, the brain–gut–microbiota axis allows for bidirectional communication, involving both nervous (peripheral and central) and endocrine and immune systems [67]. There are several potential suggestions that may explain the role of the gut flora in the pathogenesis of AD [68,69]. These include the production of neurotoxic substances such as d-lactic acid, homocysteine, pro-inflammatory cytokines and ammonia, which may cause anxiety, memory impairment and other cognitive disorders [65,70,71,72]. On the other hand, intestinal metabolites are also an important issue (more than 100 different metabolites are known) and may have pro-inflammatory effects, increasing the aggregation of amyloid and tau proteins in the brain, but a direct link between intestinal metabolites and AD pathogenesis has not been demonstrated [73,74]. The animal models used for this type of study are devoid of gut microflora. The authors showed a significant reduction in amyloid accumulation and its neurotoxicity in these animals; in contrast, after exposure of the test mice to the gut microflora of control mice, the appearance of brain tissue abnormalities was observed [61]. Clinical studies have also confirmed a difference in the intestinal microflora in AD patients compared to healthy individuals. Reduced biodiversity and a decrease in the number of *Firmicutes* [62] and *Proteobacteria* [75], as well as an increase in the percentage of *Bacteroidetes*, were observed in diseased subjects [62,75]. Quantitative abnormalities of the intestinal microflora have also been observed—an increase in *Shigella/Escherichia* (pro-inflammatory microbe) and a decrease in *Escherichia rectal* (anti-inflammatory microbe) [76]. Additionally, studies in Japanese patients with AD have found butyrate-producing bacteria, a compound that affects cognition [77]. Other studies have shown that bacterial or viral infection may also be one of the causes of AD. An association between infection with *Helicobacter pylori* [78,79], *Borrelia burgdorferi* or *Chlamydia pneumoniae* [79] and increased levels of β-amyloid peptides 1–40 and 1–42, and enhanced release of massive inflammatory mediators has been demonstrated [78,79]. Induction of hyperphosphorylation of tau protein by activation of glycogen-3β synthase kinase has been demonstrated with *Helicobacter pylori* filtrate in an in vitro study [80]. Furthermore, high levels of bacterial lipopolysaccharide have been found in the brain tissue of AD patients, which affects the inflammatory brain [81]. Viruses that may play a role in the pathogenesis of AD include *Herpes simplex* virus type 1 [82], *cytomegalovirus* (in combination with *Herpes simplex* virus type 1) [83] and *Varicella–Zoster* virus [84,85], and the most recent data indicate *hepatitis C* virus, *Epstein–Barr* virus, *human herpes* virus 8 and *human papillomavirus* [86].

Probiotics and prebiotics have been proposed following reports of a possible role for the gut microflora in AD pathogenesis. Animal studies have shown promising results. Bonfilii et al. [87] studied the effect of probiotic treatment with the SLAB 51 cocktail—“*a formulation made of nine live bacterial strains (Streptococcus thermophilus, bifidobacteria (B. longum, B. breve, B. infantis), lactobacilli (L. acidophilus, L plantarum, L. paracasei, L. delbrueckii subsp. bulgaricus, L. brevis)*”—on intestinal microflora in the mouse transgenic model (3xTg-AD mice in the early stage of AD). Probiotic therapy with the SLAB 51 cocktail increased intestinal metabolites of the short-chain fatty acid type, and these significantly impede the formation of toxic soluble amyloid aggregates. The authors observed improved cognitive function in the test animals, as well as reduction in Aβ aggregates and brain injuries and partial restoration of altered neuronal proteolytic pathway (the ubiquitin proteasome system and autophagy) [87].

Kobayashi et al. [88] evaluated the effect of oral administration of *Bifidobacterium breve* strain A1 on behavioral behaviors and physiological processes in AD model mice. The authors showed that the applied probiotic “*reversed the impairment of alternation behavior in a Y maze test and the reduced latency time in a passive avoidance test*”. Additionally, inhibition of “*hippocampal expressions of inflammation and immune-reactive genes that are induced by amyloid-β was observed*” [88]. These data indicate that applied *Bifidobacterium breve* strain A1 may potentially reduce or prevent cognitive impairment in AD [88].

Ano et al. [88] identified and evaluated the effects of dipeptides containing tryptophan-tyrosine and tryptophan-methionine on microglia activation in AD transgenic mice. They showed that compounds suppressed the microglial inflammatory response (induced by lipopolysaccharide) and enhanced the phagocytosis of Aβ. Orally administered tryptophan-tyrosine containing peptide was observed to improve cognitive function by suppressing microglia inflammation and reduced “*hippocampal long-term potential deficit, and memory impairment in aged mice*” [89].

In another study, probiotic supplementation was shown to improve cognitive function scores and reduce amyloid plaques in the hippocampus in AD transgenic mice [90]. Similar results were observed after administration of a prebiotic [91].

Another research group evaluated the effect of probiotics in rats that were induced to be disturbed by an antibiotic (ampicillin). Probiotics caused the disorder to regress [92].

Kobayashi et al. [93] conducted a randomized, double-blind, placebo-controlled trial to evaluate the effect of *Bifidobacterium breve* A1 supplementation (for 12 weeks) in older people with memory complaints. The results were evaluated using the Japanese version of the Repeatable Battery for the Assessment of Neuropsychological Status (RBANS) and the Mini-Mental State Examination (MMSE). Neuropsychological tests were performed at baseline and at the end of the study. A significant difference was observed between the study and control group in the subscale ‘immediate memory’ of RBANS and MMSE total score in the subjects with low RBANS total score at baseline. In addition, supplementation did not affect blood parameters or cause adverse effects. These results may suggest a potential positive and safe effect on cognitive function in elderly patients with memory impairment [93].

Tamtaji et al. [94] conducted a randomized, double-blind, controlled clinical trial that evaluated the effects of selenium (200 μg/day) and selenium with probiotic (containing *Lactobacillus acidophilus*, *Bifidobacterium bifidum*, and *Bifidobacterium longum*) involving patients with AD. The study was conducted over a period of 12 weeks. Selenium supplementation alone significantly reduced “*high sensitivity C-reactive protein, insulin, homeostasis model of assessment-insulin resistance (HOMA-IR), LDL-cholesterol and total-/HDL-cholesterol ratio, and significantly increased total glutathione and the quantitative insulin sensitivity check index (QUICKI)*”. On the other hand, combination supplementation (selenium + probiotic) resulted in a significant decrease in high-sensitivity C-reactive protein, and insulin concentrations and a significant increase in total antioxidant capacity, as well as a significant improvement in lipidogram results compared to both the group that received selenium alone and compared to placebo. The above results show improvements in both cognitive function and metabolic outcomes of patients [94].

These studies clearly indicate that probiotic and prebiotic therapy/prophylaxis significantly reduces memory and cognitive impairment by reducing levels of inflammatory and oxidative biomarkers [95].

## 7. Neuroinflammation in AD

Studies performed in recent years point to common points of AD and cerebral ischemia. Cognitive impairment is often found in patients after cerebral ischemic incidents [96]. The same cognitive deficits have also been shown in animal models of cerebral ischemia [97,98]. There are also a growing number of reports suggesting that ischemic damage to brain tissue promotes the neurodegenerative changes characteristic of AD. This is explained by the presence of inflammation in the brain [66], accumulation of damaged mitochondria [80], generation of reactive oxygen species [82,99,100] and amyloid protein precursor accumulation as well as tau protein dysfunction [66,101,102], resulting in neuronal damage, particularly evident in the hippocampal area. The reverse mechanism has also been demonstrated, in which Aβ and tau pathologies can promote mitochondrial defects [13,102,103]. Energy deficits and Aβ1-42 oligomers, resulting from mitochondrial dysfunction, contribute to intracellular Ca^2+^ imbalance and 5′ AMP-activated protein kinase activation. Such disturbances cause synaptotoxicity and memory loss [13,104]. On the other hand, in vitro studies indicate that tau protein disrupts axonal transport in mitochondria by blocking microtubule pathways, resulting in ATP deficiency, synapse starvation and enhanced oxidative stress. In addition, it has been noted that such disruption of axonal transport of mitochondria causes abnormal accumulation of mitochondria in large thickening dystrophic and degenerating neuritis [105]. These disorders consequently give rise to neurodegenerative changes [106]. Viewed from a physiological angle, mitochondrial quality control is regulated by the processes of mitochondrial biogenesis and mitophagy. The process of mitophagy itself is based on the removal of damaged or malfunctioning mitochondria. This happens by directing redundant mitochondria to lysosomes, where they are degraded. Mitophagy is a particularly important process and plays a role in regulating cellular homeostasis under both physiological and stress conditions. An important point is that damaged mitochondria are not repaired, and malfunctioning of these organelles causes overproduction of ROS, release of mtDNA (which can induce innate immunity), and abnormalities in release of cytochrome c and other apoptogenic factors can result in cell death [107,108]. AD patients have also been found to accumulate dysfunctional mitochondria (especially in the hippocampus) and to disrupt the mitophagy process, as confirmed by studies in animal models as well as in induced pluripotent stem cell-derived human AD neurons [13,102,109,110,111,112]. Therefore, research is being conducted on various compounds that could regulate the mitophagy process.

### 7.1. Vascular Endothelial Growth Factor (VEGF)

Liu et al. [113] evaluated VEGF in various models of AD. Genetically modified APP/PS1 mice were injected with Adeno-associated virus (AAV)-VEGF into the hippocampus and then cognitive function was assessed using the Morris water maze test and Aβ levels in the hippocampus were also measured. In another model, SH-SY5Y cells treated with Aβ25-35, cell viability and ROS levels were measured. The effects of VEGF on mitophagy, autophagy and mitochondrial biogenesis were also evaluated in vitro as well as in vivo. The authors found improved spatial learning and memory along with reduced Aβ levels. In vitro studies showed increased cell viability and decreased ROS production, indicating a protective effect against neurotoxicity. In addition, Liu et al. [113] observed improved mitochondrial structure and function, as well as increased mitochondrial number due to stimulation of mitochondrial biogenesis.

Another research group also evaluated the effects of VEGF, but in this case the study was conducted in PDGF-hAPP(V717I) transgenic mice that were administered VEGF by intraperitoneal injection for three consecutive days [114]. The authors showed that VEGF reduced memory impairment. Additionally, they showed an increase in “*CD34(+) cells in peripheral blood, vWF(+) vessels increasing in hippocampus, and CD34(+)/VEGFR2(+), vWF(+)/VEGFR2(+) and BrdU(+)/vWF(+) cells expressing in hippocampus*”. Significantly increased levels of choline acetyltransferase and reductions in Aβ accumulation were also observed [114].

### 7.2. Kisspeptin

Kisspeptin belongs to a group of neuropeptides from the family of metastasis suppressors and activates the hypothalamic–pituitary–gonadal axis. Its main function is based on the regulation of reproductive and metabolic processes. There are also reports suggesting a neuroprotective effect of the neuropeptide against Aβ toxicity [112,115].

In vitro studies (on SKNSH cell line, hippocampus explant cultures and hippocampus of aging Wistar rat models) have shown that the neuropeptide induces mitophagy and autophagy processes by “*calcium, Ca2+/CaM-dependent protein kinase kinase β, AMP-activated protein kinase, and Unc-51 like autophagy activating kinase signaling pathway that is independent of mammalian target of rapamycin”.* In turn, in vivo studies indicate an increase in mitochondrial numbers, as well as Complex I activity, and ATP levels. These results may suggest that kisspeptin shows beneficial effects on memory or cognitive dysfunction associated with mitochondrial dysfunction [112].

Ebrahimi Khonacha et al. [116] evaluated the effects of kisspeptin on learning, memory and cognitive impairment induced by injected bilaterally Aβ1-42 (2 µg/μL) or saline as a vehicle into the hippocampal CA1 area in the Morris water maze test in rats. One week later, kisspeptin (1.5 or 2 µg/µL) was injected i.c.v. before or after each training session for 3 days. Memory assessment tests were performed 24 h later. After analysis of the results, it was found that the tested compound alone significantly increased spatial memory consolidation and retrieval. Furthermore, the neuropeptide significantly alleviated Aβ-induced memory impairment [116].

## 8. Selective Serotonin Reuptake Inhibitors (SSRI)

There are numerous reports that antidepressants from the SSRI group exhibit neuroprotective effects with a diverse mechanism of action. Preclinical and clinical studies indicate beneficial effects on cognitive function [14,117,118,119,120,121,122,123]. Furthermore, literature data indicate that SSRIs “*increase neurotrophic factors including brain-derived neurotrophic factor, promote neurogenesis in the hippocampus, and reduce levels of toxic Aβ*” [124]. There are also reports of properties to decrease Aβ generation and plaque load. Therefore, due to its interesting properties, the SSRI group of drugs is increasingly becoming the focus of research into the treatment of patients with AD. An additional aspect is the fact that depression itself represents an increased risk of AD, and therefore the applied treatment could significantly delay the progression to AD [122].

### 8.1. Citalopram

Citalopram is a selective serotonin reuptake inhibitor that, according to scientific reports, may have neuroprotective effects in AD patients. Reddy et al. [14] evaluated the effect of citalopram on mitochondrial dysfunction in immortalized mouse primary hippocampal cells (HT22) expressing APP (SWI/IND) mutations. Exposed cells showed “*reduced levels of the mitochondrial fission genes, increased fusion, biogenesis, autophagy, mitophagy, and synaptic genes*” compared to the control group, which showed increased mRNA levels of mitochondrial fission genes and changes indicative of impaired autophagy and mitophagy. Furthermore, it was shown that cells in the experimental group had an increased number of mitochondria and cell survival rates were increased. These results may suggest a protective role for citalopram against damage associated with Aβ accumulation in AD patients [14]. Clinical trials evaluating the effects of citalopram have also been conducted. The first of these, the Citalopram for Agitation in Alzheimer Disease Study, was a randomized, placebo-controlled, double-blind, parallel group trial in which participants were patients with probable AD and clinically significant agitation. Study participants received citalopram or placebo for 9 weeks. Dosing started at 10 mg/day and was titrated to 30 mg/day over 3 weeks. The results show that the drug reduced patients’ agitation, but caused cognitive impairment and cardiovascular side effects, which limits its practical use in patient therapy [117].

Another clinical study evaluated the effect of citalopram on, among other things, cognitive function in patients with moderate AD and clinically significant behavioral and psychological symptoms of dementia. Study participants (study group) received memantine (at the dose of 20 mg/day) plus citalopram (starting at the dose of 10 mg/day to titrated to 30 mg/day over 2 weeks). The control group received memantine (at the dose of 20 mg/day) plus placebo. The whole study lasted for 12 weeks. Analysis of the results showed that the overall neuropsychiatric scores of the patients after treatment were significantly lower compared to the pre-treatment scores in both groups. However, indices assessing symptoms such as apathy, dysphoria and anxiety obtained lower values. Among the adverse effects, as in the previous study, only QTc interval prolongation was found in two patients treated with the combination with citalopram at a dose of 30 mg/day. In conclusion, the drug combination can significantly improve cognitive function as well as reduce behavioral and psychological symptoms in patients with moderate AD, but a dose <30 mg/day is recommended [118].

### 8.2. Escitalopram

Another drug being considered as a candidate for the treatment of AD is escitalopram, the most specific selective serotonin reuptake inhibitor [119].

The authors evaluated the effect of chronic administration of the drug on brain interstitial fluid Aβ and Aβ plaque size in the amyloid precursor protein (APP)/presenilin 1 mouse model of AD (APP/PS1+/− hemizygous mice to wild-type C3H/B6 mice). Animals received the drug at doses of 2.5 or 5 mg/kg or vehicle (2% DMSO in normal saline), injected intraperitoneally for 28 days or in a single dose. Analysis of the results showed that escitalopram administered in the acute model reduced brain interstitial fluid Aβ by 25% by increasing α-secretase cleavage of APP. Interesting results were also obtained in the chronic model, with a significant reduction in plaque load. At a dose of 2.5 mg/kg, a 28% reduction was achieved, whereas at a dose of 5 mg/kg, the reduction was 34%. Furthermore, the authors found that escitalopram at a dose of 5 mg/kg, although not removing existing plaques, stopped their growth [119].

Sheline et al. [120] recently published the results of a double-blind placebo-controlled clinical trial on escitalopram. The study was conducted in healthy older volunteers who were then allocated to the following groups: placebo, 20 mg escitalopram × 2 weeks, 20 mg escitalopram × 8 weeks, or 30 mg escitalopram × 8 weeks. Both before and after treatment, CSF samples were collected from participants. Analysis of the results showed an overall 9.4% greater reduction in CSF Aβ42, which may suggest an interesting therapeutic option in studies involving AD patients [120].

Among the new data, there are also reports that escitalopram may affect tau hyperphosphorylation. In vitro studies have shown that exposure of human embryonic kidney HEK293/tau441 cells (were pretreated with 4 μM of forskolin for 2 h) to the antidepressant (at concentrations of 0, 5, 10, 20, 40, 80 μM) for 22 h, “*protect tau from hyperphosphorylation induced by pharmacological activation PKA at a dose of 20, 40, and 80 μM in vitro*” [125].

Similar results were obtained by another group [126] who showed that escitalopram inhibited Aβ1-42-induced tau hyperphosphorylation in primary hippocampal neurons [125].

Wang et al. [124] evaluated a possible mechanism of action of this drug in aged P301L mice. Animals were intraperitoneally injected with escitalopram for a period of 4 weeks. In the next stage of the experiments, behavioral tests were performed and brain tissue was collected for analysis. Based on the results of the behavioral tests, no significant improvement in learning and memory processes was observed in the ageing mice. On the other hand, analysis of the brain tissue of the animals showed significantly decreased tau phosphorylation, which may suggest some protective properties of escitalopram [124].

## 9. Natural Products and Alzheimer’s Disease

### 9.1. Resveratrol (RV)

RV (3,5,4′-trihydroxystilbene) is a compound from the polyphenol family that occurs naturally, mainly in black grapes, but also in blackberries or peanuts. There are many data on the promising properties of RV, including anticancer, antioxidant, cytoprotective, anti-inflammatory (including inhibition of microglial activation and the regulation of neuroinflammation), neuroprotective or anti-aging [127,128,129,130]. Previous studies indicate that RV may be a preventive or therapeutic option for patients with disorders such as insulin resistance, diabetes, lipid disorders and obesity or cardiovascular disease [131,132].

More recent studies indicate that RV has properties that may influence various pathomechanisms of AD. These range from molecular disorders, including processes responsible for the accumulation of abnormal proteins. One of the most important pathways whose disruption affects the development of AD is the ubiquitin-proteasome system, which is the primary proteolytic mechanism to aberrant clearance proteins, including Aβ and p-Tau [133]. Studies on AD models indicate that RV improves proteosome functionality in AD models. The polyphenol has been shown to increase Aβ clearance on the one hand, and to decrease protein production on the other by stimulating proteasomal proteolysis, as demonstrated in cell lines expressing APP695 and an Alzheimer’s model of *Caenorhabditis elegans* [134,135,136].

Other studies have shown that RV reduces mitochondrial dysfunction. This compound has been observed to exhibit anti-inflammatory, antioxidant and anti-apoptotic properties [137]. Moreover, it induces autophagy and mitophagy, which was confirmed in an in vivo model [138].

In several in vivo studies, RV has been shown to inhibit ROS formation by suppressing nicotinamide adenine dinucleotide phosphate oxidase [139] and promoting the expression of antioxidant enzymes including superoxide dismutase, catalase, thioredoxin, and glutathione peroxidase [140].

Wang et al. [141] suggested that RV (at a dose of 200 mg/kg/day for 8 weeks) could be an option for AD-adjuvant therapy after human umbilical cord stem cell transplantation. This was due to the fact that RV stimulated factors such as the expression of brain-derived neurotrophic factor precursor, neuronal growth factor, and neurotrophin 3, which physiologically regulate the process of neurogenesis and also affect memory and learning [141]. Another research group, Simão et al. [142], injected RV intraperitoneally (at a dose of 30 mg/kg for 7 days) into rats. Then, the rodents were subjected to 10 min of four-vessel occlusion. Analysis of the results showed that RV prophylaxis reduced astrocyte and microglia activation and suppression of the inflammatory response in the hippocampus [142]. Moussa et al. [143] conducted a study evaluating the effect of RV in patients with mild-moderate AD. The authors showed that RV reduced pro-inflammatory factors and increased activation of microglia/macrophages was observed, which may indicate a neuroprotective effect through induction of long-term adaptive immunity. Again, analysis of the results revealed a significant reduction in brain volume (excluding CSF, brain stem, and cerebellum) and an increase in ventricular volume. The above changes did not correlate with greater cognitive decline. These changes are most likely related to the strong anti-inflammatory effect in the brain of the patients and the reduction in tissue swelling, as no potential neuronal loss was excluded. Furthermore, no significant side effects were found in the study, confirming the safety and good tolerability of the polyphenol [143].

Another group conducted a randomized, double-blind, placebo-controlled phase II study of resveratrol in the treatment of mild to moderate dementia due to AD [144,145]. They evaluated the safety and tolerability of RV and its effects on biomarkers of this neurodegenerative disease. Low nanomolar concentrations were determined in CSF and, by implication, in brain tissue, indicating the low bioavailability of oral RV. The next step was to determine the levels of anti-inflammatory and pro-inflammatory cytokines, chemokines and metalloproteinases in plasma and CSF samples. A significant, approximately 50% decrease in the level of metalloproteinase-9 in CSF was observed. Similar results were also obtained in other in vivo studies (Wistar rats) [146] and in the already mentioned clinical trial [143]. This is a particularly important result because metalloproteinase-9 affects BBB permeability and “*cleaves the vascular basal lamina and tight junctions in the neurovascular bed*” [145,147]. The above data may indicate that RV, by decreasing BBB permeability, may limit infiltration of inflammatory cells and mediators [145,147].

Other studies have shown that RV may modify the composition of the gut microbiota, which, as previously mentioned, may be important for preventive treatment of AD patients. There are few data on this topic, but it has been suggested that RV produce beneficial metabolic effects through interactions with the gut microbiota [148].

RV has been shown to be a potent activator of silent information regulator-1 (SIRT1). Sirtuins are “*deacetylases that link energy balance (NAD^+^/NADH) to regulation of gene transcription*” [145]. It has been shown that reduced levels of SIRT1 expression exacerbate, in turn, over-expression of SIRT1 reduces Aβ production [138,149,150]. SIRT1 has also been shown to regulate other processes that may be involved in AD pathogenesis, i.e., cellular homeostasis, mitochondrial biogenesis, glucose metabolism as well as tissue insulin sensitivity, all of which directly or indirectly affect mitochondrial survival [151,152,153]. In addition, RV also increases cerebral blood flow, promotes neurogenesis and prevents hippocampal damage, and by acting on SIRT1, RV improves synaptic pathway plasticity and cognitive function [154].

In conclusion, RV affecting the regulation of so many processes including, but not limited to, antioxidant, anti-inflammatory, neuroprotective or anti-apoptotic effects may represent a promising, safe and well-tolerated therapeutic option for AD patients.

### 9.2. Curcumin

Curcumin is the main polyphenol found in the turmeric curry (*Curcuma longa*). This compound has been found to have strong beneficial properties affecting cognitive function or memory, among other things. Furthermore, the polyphenol has been shown to pass through the BBB [155]. This may be related to the fact that the main biosynthetic pathway of turmeric begins with phenylalanine, which in turn is a precursor in the biosynthesis of flavonoids, compounds that have been proven to have therapeutic effects in neurodegenerative disorders [156,157]. In addition, curcumin and its derivatives are among the most bioactive components of saffron, which boasts many properties such as anti-inflammatory, antidiabetic, antiviral, antiproliferative, antioxidant, pro-apoptotic as well as anti-amyloidogenic effects [158,159].

More recent publications indicate that curcumin also affects Aβ plaque aggregation and tau protein hyperphosphorylation [155,160]. Small et al. [160] conducted a double-blind, placebo-controlled 18-month trial in which they evaluated the effects of curcumin (90 mg, twice daily) in adults without dementia. After analyzing the results, the authors found improvements in verbal and visual memory, attention in the study group, while imaging studies found significantly decreased tau accumulation in the amygdala and in brain regions modulating mood and memory [160].

Zheng et al. [161] evaluated the effect of applied curcumin (at doses of 150 or 300 mg/kg/day, intragastrically, for 60 days) in 5×FAD transgenic mice as an AD model. After the experiments, a significant decrease in Aβ production was demonstrated, which is most likely related to a decrease in β-secretase 1 expression [161,162]. Furthermore, a reduction in synaptic degradation, as well as improving spatial learning and memory impairment, was observed [161]. On the other hand, Xiong et al. [163] showed that curcumin affected the protein levels of presenilin-1 and glycogen synthase kinase-3β (GSK-3β). These proteins, together with protein in the γ-secretase complex, are involved in Aβ production. When the authors treated human neuroblastoma SHSY5Y cells with curcumin, a significant decrease in Aβ production was observed, as well as a decrease in PS-1 and GSK-3β proteins [163]. Similar promising results were obtained by another research group that evaluated the effects of curcumin (80mg/kg/day, orally, for 3 months) in a rat model of AD disease. Aβ accumulation in the hippocampus was shown to be reduced, cognitive function improved, and a significant reduction in apoptosis and oxidative stress processes was observed [164].

Another important finding is the demonstration that curcumin inhibits aggregation and promotes disaggregation of fibrillar Aβ, which has been confirmed in both in vivo and in vitro studies [155,165,166].

In addition, other studies have shown that curcumin regulates the insulin signaling pathway and thus affects cognitive dysfunction associated with the already mentioned insulin resistance. Feng et al. [167] tested the mechanism of curcumin action in a transgenic mouse model of AD. Curcumin was administered to the animals for a period of 6 months. After this period, it was shown that “*the hippocampal CA1 tissue expressed lower levels of insulin receptor and IRS-1, while the expression of PI3K, phosphorylated PI3K, Akt, and phosphorylated Akt increased compared to control*”. The above data confirmed that curcumin stimulated the PI3K/Akt signaling pathway, resulting in reduced insulin resistance [167]. Other authors additionally showed that curcumin therapy improved memory in a rat model of diabetes [168].

Long-term studies have shown other equally important properties of the compound. The polyphenol lowers cholesterol, binds copper, inhibits neuroprotective effects by reducing the inflammatory activity of microglia, and has also been shown to increase the phagocytic activity of microglia [169]. The great variety of effects as well as the properties of curcumin allow multidirectional action and modification of the disease process [170]. Candidate drugs and compounds for the management of AD are shown in Table 2 and Table 3.

## 10. Current Clinical Trials

As already mentioned, it has not yet been possible to obtain the ‘‘ideal drug’’ for AD patients, which would not only reduce symptoms but also influence the pathomechanism of the disease. Clinical trials have been ongoing for a long time to gain a better understanding of the disease, its pathomechanisms, both those already known and those not yet known, and to search for new drug candidates for AD therapy. Currently, a large number of clinical trials are being conducted at various stages of progression. The following is a brief description of selected compounds involved in the clinical trials and presented in Table 4.

### 10.1. BIIB092

In 2018, the results of a Phase I clinical trial evaluating the safety and tolerability of BIIB092 were released. BIIB092, as a humanized monoclonal antibody, binds to the N-terminal region of tau. Healthy participants received single doses ranging from 21 to 4200 mg. Based on the results, the compound was found to be safe and well tolerated. Furthermore, “*serum BIIB092 concentrations increased in a dose-proportional manner and suppressed unbound cerebrospinal fluid N-terminal tau by 67–97% at 28 days after dose, with doses of ≥210 mg producing persistent unbound N-terminal tau suppression over 12 weeks*” [177]

### 10.2. GV1001

The available data indicate that GV1001 inhibits neurotoxicity, apoptosis and reactive oxygen species generation induced by Aβ in neural stem cells “*by mimicking the extratelomeric functions of human telomerase reverse transcriptase*”. In studies using animal models, GV1001 has been shown to improve cognitive function and memory, as well as significantly reduce Aβ and tau protein [173].

### 10.3. AR1001

According to the available data, AR1001 shows safety, great potential and favorable effects for the central nervous system. Furthermore, the compound was shown to have good pharmacokinetics, bioavailability and penetration through the BBB [174].

### 10.4. CT1812

As a novel allosteric antagonist of the sigma-2 receptor complex, CT1812 prevents and displaces Aβ oligomer binding to neurons. This compound has been shown to alleviate subsequent synaptotoxicity and restore cognitive function, as demonstrated in ageing transgenic mouse models of AD. In addition, the results of a phase I clinical trial evaluating the safety of use and the occurrence of possible side effects in both healthy young (<65 years) and elderly (65–75 years) subjects were released in 2019. The doses used were 10 to 1120 mg in phase A (a single dose), and 280, 560 and 840 mg once daily for 14 days in phase B, while in elderly subjects, a dose of 560 mg was administered once daily for 14 days, respectively. The drug was found to be well tolerated in all study groups, without major side effects (mild to moderate headache and gastrointestinal symptoms). For older participants, no significant differences in cognitive function were observed compared to before the study [178].

### 10.5. Montelukast

Another drug being evaluated is montelukast. This drug is used in the treatment of inflammatory respiratory diseases, including bronchial asthma. Previous studies have shown that montelukast has beneficial effects on inflammation in the brain and on neuronal damage, BBB integrity and Aβ accumulation [176].

## 11. Conclusions

AD as a devastating neurodegenerative disease negatively affecting cognition and causing evident functional decline can be difficult to cure with the existing pharmacological approach. Evidently, the drugs available on the market (for instance, acetylcholinesterase inhibitors or memantine) are in fact symptomatic, offering only transient improvement in cognition or delaying the progress of the disease [179]. Consequently, effective drugs alleviating cognitive decline and behavioral disturbances in AD patients are intensively searched for.

One strategy assumes a search among drugs already approved for other conditions, and a good example is insulin. Obviously, this strategy is considerably less time-consuming than a strategy seeking novel drugs among investigational compounds. Data on the use of intranasal insulin are encouraging. In fact, insulin is an enhancer of memory performance and this effect is likely to be associated with its positive influence on brain synaptic plasticity, neuronal glucose uptake and neuropathological consequences of AD [51]. After all, the beneficial activity of intranasal insulin may be closely related to the normalization of dysfunctional glucose metabolism, which probably results from genetic risk factors in AD patients [180]. In addition, some antidepressant drugs (citalopram, escitalopram) were shown to be clinically beneficial in at least some aspects of AD patients. Very recent clinical data indicate that antidepressants (citalopram, escitalopram, sertraline, mirtazapine), especially when combined with antipsychotic drugs (quetiapine, risperidone, olanzapine, levomepromazine), significantly reduced neuropsychiatric symptoms (agitation and/or aggression) in AD patients [181]. The authors of this study are aware of a number of limitations—the patients were not randomized and treatment adherence was not controlled. Furthermore, the patients were not evaluated in terms of the QTc interval or electrolytic disturbance, which may be expected with the use of antidepressants and antipsychotics [181]. No doubt, both antidepressants and antipsychotics must be used with caution in the elderly population in order to avoid serious adverse effects. Bexarotene is another approved drug bearing curative potential in the treatment of AD. Another drug from this group is alitretinoin [182]. The results of experimental studies are generally encouraging but limited clinical data are not that optimistic, mainly due to the resistance of apoE4 carriers and adverse activity. The adverse effects of retinoid receptor agonists are likely to depend on the lack of their affinity to subtypes of these receptors [182]. Perhaps the development of newer selective agonists in this group of compounds will be a proper solution to this problem [182]. Last but not least, probiotic drugs must be mentioned in terms of AD management, as they seem to be promising adjuvant agents. Probiotic-fermented milk supplementation was applied for 90 days in AD patients with cognitive impairment [183] and the patients were considerably improved in terms of memory, visual-spatial/abstraction abilities and executive/language functions. Moreover, inflammatory and oxidative stress markers in plasma were significantly lowered when compared to the results obtained before probiotic supplementation [183].

There is extensive evidence pointing to a close relationship between brain ischemia and AD [66,184]. Amyloid hypothesis assumes aberrant upregulation of Aβ production and/or its reduced clearance, which results in its accumulation in the brain. A crucial point is whether Aβ causes neurodegeneration, or whether neurodegeneration itself is responsible for Aβ accumulation [66]. A possibility arises that episodes of brain ischemia initiate a cascade of harmful events, leading to neurodegeneration, Aβ accumulation, tau protein pathology and AD. Moreover, brain ischemia disturbs the expression of Alzheimer genes (amyloid-processing, amyloid precursor protein or tau protein genes), eventually causing the brain alterations characteristic of sporadic AD [66,185]. The above data on the possible prominent role of brain ischemia in the induction of Alzheimer-type dementia may open up new innovative (probably preventive) treatments of AD with candidate drugs. One such candidate might be myricetin (a polyphenol) [186], whose beneficial characteristics seem perfectly tailored in this aim. As a matter of fact, myricetin reduces Aβ and amyloid oligomers/fibrils production, diminishes neuroinflammation, expresses powerful antioxidant activity and elevates brain acetylcholine level. Another compound with antioxidative and anti-inflammatory properties, curcumin, shares similar promising potential [187]. In addition, resveratrol, as a antioxidative and anti-inflammatory compound, has to be considered in this regard. On the basis of the available data, it can be concluded that the beneficial effects of these natural compounds are related to a number of different mechanisms. RV acts as an antioxidant [127,128,129,130,137,139], but can also directly affect Aβ formation and accumulation by acting on the ubiquitin-proteasome system [133,134,135,136] as well as by activating SIRT1 [138,145,149,150]. The situation is similar for curcumin. The compound also exhibits antioxidant effects as well as direct anti-amyloidogenic effects [158,159]. The latter is due to various mechanisms, including curcumin’s effects on Aβ plaque aggregation (inhibits aggregation and promotes disaggregation of fibrillar Aβ) and tau protein hyperphosphorylation [155,160,165,166], as well as the regulation of protein levels of presenilin-1 and GSK-3β [163]. What is particularly important is that all compounds presumed to exert desirable effects on post-ischemic events may be easily accessible and are not expensive. Certainly, these compounds, apart from their beneficial effects on post-ischemic neurodegeneration, have been proved to exert direct actions counteracting the AD pathology.

Out of investigational drugs, CN-105 seems to have significant potential. On one hand, its effects in the mouse model of AD pointed to reduced Aβ pathology and cognitive deficits evaluated in a Morris water maze and contextual fear conditioning [32]. In addition, the drug exhibited a remarkable safety profile and is currently being introduced into a clinical trial devoted to postoperative dementia. The results of this study will probably have a crucial predictive value for its further evaluation in AD patients. Another example of an investigational compound might be PO21, which is the most active fragment of the ciliary neurotrophic factor. To reduce its susceptibility to exopeptidases and enhance its brain penetration through the BBB, the compound was admantylated on its C-terminal. PO21 exerted a number of beneficial effects—it inhibited tau and Aβ pathologies due to its inhibitory influence on GSK-3β [188]. Its clear-cut neurotrophic activity may also account for its beneficial effects in an animal model of AD [189]. This probably indicates that reduced neurotrophic signaling during development may participate in the etiology of AD [189].

Although the initial clinical studies with BACE1 inhibitors are disappointing, according to Hampel et al. [11], the enzyme may be still regarded as a promising therapeutic target in AD. Hopefully, some more compounds with high substrate selectivity will emerge showing beneficial activity in particular patient population and/or disease stage [11].

Only some novel therapeutic approaches (some with initial clinical background) have been reviewed. There are other pharmacological options to be examined, such as β-lactam antibiotics [190]. It is of particular importance that some innovative therapies (5-HT_6_ receptor antagonists, the M_1_-positive muscarinic allosteric modulator MK-7622, and xanamen—an 11-β-hydroxysteroid dehydrogenase antagonist) have failed to improve cognition following clinical evaluation [191]. AD patients, apart from cognitive deficits, also exhibit neuropsychiatric symptoms, and according to Cummings [191], novel therapies seem more successful compared to cognitive impairment. Good examples included pimavanserin, which is effective against dementia-related psychosis, and suvorexant, which is effective against AD insomnia [191].

It seems that multitarget therapy, as recommended by Guzman-Martinez et al. [192], as well as taking preventive measures (proper nutrition, nutraceutics, exercising, active social and mental life, avoidance of toxic substances) may be recommended to AD patients or persons at risk of AD. Certain drugs (for instance, these enhancing neurotrophic signaling) would have to be implemented at early stages of AD. In this aim, reliable blood biomarkers are necessary, and a recent study by Corbo et al. [193] offers such a possibility, but their results have to be confirmed in other studies.

## Figures and Tables

**Table 1:** Pharmacologic therapies for the management of Alzheimer disease.

	Drugs	Dosage of the Medicine	Adverse Effects	Metabolism	References
**Cholinesterase inhibitors**	Donepezil (Aricept)	5 mg orally at bedtime for 4 to 6 weeks, then 10 mg orally at bedtime; can increase to 23 mg at bedtime after 3 weeks at 10-mg dose	Atrioventricular block, decreased appetite, diarrhea, dizziness, headache, hypertension, nausea, syncope, torsades de pointes, vomiting, weight loss	CYP2D6, CYP3A4	[3,4,8]
	**Galantamine (Razadyne)**	Immediate release: 4 mg orally 2 times per day for 4 weeks, then increase to 8 mg 2 times per day for 4 weeks, then 12 mg 2 times per day Extended release: 8 mg orally per day for 4 weeks, then increase to 16 mg per day for 4 weeks, then 24 mg per day When switching from oral to transdermal administration, if total daily dosage of galantamine is less than 6 mg, use 4.6-mg patch. If total daily dosage is 6 to 12 mg, use 9.5-mg patch	Atrioventricular block, decreased appetite, diarrhea, dizziness, headache, nausea, vomiting, weight loss	CYP2D6, CYP3A4	[3,4,8]
	**Rivastigmine (Exelon)**	For Alzheimer disease: 1.5 mg orally 2 times per day for 2 weeks, then increase each dose in 1.5-mg increments every 2 weeks as tolerated to maximal dosage of 12 mg per day. Transdermal patch (for Alzheimer disease and Parkinson disease dementia): 4.6-mg patch every 24 h for 4 weeks, then 9.5-mg patch every 24 h for 4 weeks, then 13.3-mg patch every 24 h	Abdominal pain, atrial fibrillation, atrioventricular block, decreased appetite, diarrhea, dizziness, headache, myocardial infarction, nausea, vomiting	Non-hepatic	[3,4,8]
**N-methyl-D-aspartate receptor antagonist**	**Memantine (Namenda)**	Immediate release: 5 mg orally per day for 1 week, then 5 mg 2 times per day for 1 week, then 10 mg every morning and 5 mg every night for 1 week, then 10 mg 2 times per day Extended release (approved but not yet available): 7 mg orally per day for 1 week, then increase in 7-mg increments every week to target dosage of 28 mg per day	Confusion, constipation, diarrhea, dizziness, vomiting; rarely, cerebrovascular event or acute kidney injury	Non-hepatic	[3,4,8]
**Combination drug and vitamin E**	**Memantine/donepezil (Namzaric)**	7 mg/10 mg orally at bedtime for 4 weeks, then increase by 7 mg/10 mg every week as tolerated to target dosage of 28 mg/10 mg every night	Decreased appetite, diarrhea, heart block, syncope, vomiting		[3]
	**Vitamin E**	1000 IU orally 2 times per day	Hemorrhage (including cerebral); may increase all-cause mortality		

**Table 2:** Selected drugs and compounds as candidates for the management of Alzheimer’s disease.

Compounds/Drugs	Probable Mechanism of Action	Type of Research			Effects	References
		Clinical Trials	Animal Model of Alzheimer’s Disease	In Vitro		
Bexarotene	↓ ABCA1, ABCG1 expression ↑ ApoE lipidation		+		↓ Soluble or insoluble Aβ ↓ Aβ plaques Improved memory ↑ HDL levels	[19]
					Improved cognitive function Improved baseline synaptic transmission and synaptic plasticity ↓ Astrogliosis and reactive microglia in both cortex and hippocampus ↑ Expression of APOE (limited to CA1 hippocampal)	[21]
					↓ Memory deficits ↓ Interstitial fluid Aβ level No effect on amyloid deposition	[22]
					↓ Soluble Aβ40 No effect on plaque burden that exhibit Aβ amyloidosis	[23]
		+			↑ ApoE concentrations by 25% No effect on Aβ peptide metabolism Adverse reactions (non-significant)	[24]
					No effect on amyloid burden in apoE4 carriers Significant correlation between ↑ serum Aβ1-42 and ↓ in brain amyloid in apoE4 noncarriers, significant adverse reactions	[25]
ADCS-6253	Directly activates ABCA1 expression		+		APOE4 knock-in only ↑ ApoE4 lipidation ↑ ABCA1 expression ↓ Aβ and phosphorylated tau	[26]
HJ6.3–monoclonal antibody against apoE	Blocking apoE and Aβ interaction		+		↓ Soluble and insoluble Aβ ↓ Microglia ↓ Brain Aβ plaques ↑ Plasma Aβ ↓ Pro-inflammatory cytokines	[30]
HAE-4	Blocking apoE and Aβ interaction		+		↓ accumulation of Aβ in the brain	[31]
CN-105	ApoE mimetic		+		↓ Soluble Aβ ↓ Aβ plaques Improved memory	[32]
Anti-APOE antisense oligonucleotides	Silencing APOE		+		↓ Soluble APOE ↓ Soluble and insoluble Aβ ↓ Aβ plaques ↓ Dystrophic neurites	[36]
Intranasal insulin	Reduced AHP		+		↓ Cognitive impairment, improves memory in	[45]
		+			patients without (epsilon4-,) improves verbal memory	[47]
Liraglutide	Glucagon-like peptide-1 analog Activation of protein kinase A (PKA) signaling pathway		+		Prevented the “*loss of brain insulin receptors and synapses, and reversed memory impairment*” induced by AβOs ↓ AD-related insulin receptor ↓ Synaptic and tau pathologies in specific brain regions	[52]
Probiotic therapy with the SLAB 51 cocktail	↑ Intestinal metabolites of the short-chain fatty acid type		+		Impede the formation of toxic soluble amyloid aggregates ↑ Cognitive function ↓ Aβ aggregates ↓ Brain injuries Partial restoration of altered neuronal proteolytic pathway	[87]
Dipeptides of tryptophan-tyrosine and tryptophan-methionine	Suppression of the inflammatory response of microglia ↑ Aβ phagocytosis		+		Improve cognitive function ↓ Hippocampal long-term potential deficit ↓ Memory impairment	[89]
Selenium or selenium with probiotic	Anti-inflammatory and antioxidant effects ↑ Total glutathione and the quantitative insulin sensitivity check index (QUICKI)	+			↓ High sensitivity C-reactive protein Improvement of lipidogram results ↑ Cognitive function	[94]
VEGF	Anti-inflammatory effects		+		Improved spatial learning and memory along with ↓ Aβ levels	[113]
				+	↑ cell viability ↓ ROS production Improved mitochondrial structure and function ↑Number of mitochondria ↑ Stimulation of mitochondrial biogenesis	
			+		↓ Memory impairment ↑ Levels of choline acetyltransferase ↓ Aβ accumulation	[114]
Kisspeptin	Activates the hypothalamic-pituitary-gonadal axis			+	Induces mitophagy and autophagy processes	[112]
			+		↑ Number of mitochondria ↑ Complex I activity ↑ ATP levels	
			+		↑ Spatial memory consolidation and retrieval Alleviated Aβ-induced memory impairment	[116]
Citalopram	Selective serotonin reuptake inhibitor			+	↓ Levels of the mitochondrial fission genes ↑ Fusion, biogenesis, autophagy, mitophagy, and synaptic genes ↑ Number of mitochondria and cell survival rates	[14]
		+			↑ Cognitive impairment Cardiovascular side effects	[117]
Escitalopram			+		↓ Aβ level in CSF	[119]
	Selective serotonin reuptake inhibitor				↓ Plaque load Inhibition of amyloid plaque growth	
		+			↓ Aβ42 level in CSF	[120]
				+	Inhibition of tau hyperphosphorylation	[125,126]
Resveratrol	Stimulating proteasomal proteolysis Stimulating factors such as expression of brain-derived neurotrophic factor precursor, neuronal growth factor, and neurotrophin 3 Anti-inflammatory, antioxidant and anti-apoptotic action Induces autophagy and mitophagy Activator of silent information regulator-1 (SIRT1)		+		↓ Astrocyte and microglia activation and suppression of the inflammatory response in the hippocampus	[134,135,136,142]
		+			↓ MMP-9 levels in CSF ↓ Aβ40 levels in CSF ↑ Macrophage-derived chemokine, fibroblast growth factor-2 and interleukin (IL)-4 levels ↓ Plasma concentrations of pro-inflammatory mediators, including IL-1r4, IL-12P40, IL-12P70, and TNF-α ↓ Brain volume (excluding CSF, brain stem, and cerebellum) ↑ Ventricular volume	[143]
		+			↓ MMP-9 levels in CSF	[144,145]
Curcumin	Affects Aβ plaque aggregation and tau protein hyperphosphorylation Antioxidant, anti-inflammatory, antidiabetic, antiviral, antiproliferative, antioxidant, pro-apoptotic as well as anti-amyloidogenic action Regulates levels of PS-1 and GSK-3β	+			Improvements in verbal and visual memory ↓ Tau accumulation in the amygdala	[160]
			+		↓ Aβ production ↓ Synaptic degradation Improving spatial learning ↓ Memory impairment	[161]
			+		Improving cognitive function ↓ Apoptosis and oxidative stress processes ↓ Aβ accumulation	[164]
				+	↓ Aβ production	[163]

↓—decrease; ↑—increase; AHP—Ca^2+^-dependent hippocampal after hyperpolarization; ATP—adenosine triphosphate; VEGF—Vascular endothelial growth factor; GSK-3β—glycogen synthase kinase-3β; PS-1—presenilin-1; MMP-9—matrix metalloproteinase-9.

**Table 3:** Candidate drugs in selected clinical trials.

Drugs/Substances	Dosage	Time-Dependent Therapy	Route of Administration	Diagnostic Tool/Tests	Patients	References
Bexarotene	225 mg or placebo twice daily	For 5 days	Oral	Applied “*stable isotope labeling kinetics (SILK-ApoE and SILK-Aβ) to measure the effect of bexarotene on the turnover rate of apoE and Aβ peptides and stable isotope spike absolute quantitation (SISAQ) to quantitate their concentration*s” in CSF	Healthy volunteers; aged 21 to 49 years (average 32 years old); with APOE ε3/ε3 genotype	[24]
Intranasal insulin	10, 20, 40, or 60 IU or placebo five times a day	Cognition was tested 15 min after treatment and blood was drawn immediately after insulin/placebo administration and 45 min after treatment	Intranasal	Verbal declarative memory measures (Story Recall and Hopkins Verbal Learning Test) A test of selective attention (Stroop Color-Word test) A visual working memory measure (Self-Ordered Pointing Task) A test of psychomotor processing speed (Digit Symbol)	Participants with (epsilon4+or epsilon4-) the APOE- epsilon4 allele with memory-impaired with either probable AD or amnestic MCI or multiple domain MCI with amnestic features (mean age of about 77) and cognitively normal older (epsilon4+or epsilon4-) as control groups (mean age of about 74)	[47]
Selenium or selenium with probiotic	Selenium (200 μg/day) plus probiotic (containing *Lactobacillus acidophilus, Bifidobacterium bifidum, and Bifidobacterium longum*) (2 × 109 CFU/day each), selenium (200 μg/day) or placebo	For 12 weeks	Oral	Cognition was tested using the Mini-Mental State Examination (MMSE) biomarkers of inflammation and oxidative stress, metabolic profiles and plasma glucose	Patients with AD (aged 55 to 100 years)	[94]
Citalopram	Dosing began at 10 mg/day with planned titration to 30 mg/day over 3 weeks based on response and tolerability or placebo	Psychosocial intervention plus either citalopram or placebo for 9 weeks	Oral	Assessment of agitation, hostility/uncooperativeness, and disinhibition—Neurobehavioral Rating Scale agitation subscale (NBRS-A) the modified Alzheimer Disease Cooperative Study-Clinical Global Impression of Change (mADCS-CGIC) Cohen-Mansfield Agitation Inventory (CMAI) Neuropsychiatric Inventory (NPI) Activities of daily living (ADLs) Caregiver distress; cognitive safety (MMSE)	Patients with probable AD and clinically significant agitation	[117]
Escitalopram	20 mg or 30 mg/day or placebo	For 2 or 8 weeks	Oral	Lumbar punctures to sample CSF levels before and after treatment	Cognitively normal older adults (aged 50 to 84 years)	[120]
Resveratrol	up to 1 g by mouth twice daily (500 mg once daily (with a dose escalation by 500-mg increments every 13 weeks, ending with 1000 mg twice daily)	For 52 weeks	Oral	“M*agnetic microspheres internally coded with two fluorescent dyes to measure markers of neurodegeneration (Millipore, Cat#: HNABTMAG-68K)*”	Patients with mild-moderate AD	[143,144,145]
Curcumin	90 mg or placebo twice daily—(180 mg/day)	for 18 months	Oral	Verbal (Buschke Selective Reminding Test [SRT]) Visual (Brief Visual Memory Test-Revised [BVMT-R]) Memory attention (Trail Making A) Assessment of amyloid and tau accumulation in the brain (2-(1-{6-[(2-[F-18]fluoroethyl)(methyl)amino]-2-naphthyl}ethylidene)malononitrile positron emission tomography (FDDNP-PET)	Middle-aged and older adults without dementia (age 51 to 84 years)	[160]

CSF—cerebrospinal fluid; MCI—mild cognitive impairment.

**Table 4:** Currently conducted, selected clinical trials.

ClinicalTrials.gov Identifier	Type of Clinical Trial	Phase	Compounds/Drug Tested	Dosage/Route of Administration	Characteristics of the Clinical Trial	Characteristics of the Patients		References
						Age of the Patient	Diagnosis	
NCT04308304	A randomized, double-blind, placebo-controlled study	I	MK-1942-005 or placebo	8 mg to ≤ 50 mg/orally	Evaluation of the safety and pharmacokinetics of MK-1942 and donepezil when co-administered to participants Evaluation of whether the combination increases the incidence or severity of adverse events	50 to 85 years	With AD with mild-to-moderate cognitive impairment stably treated with donepezil.	[171]
NCT03352557	A randomized, double-blind, placebo-controlled, parallel-group study	II	BIIB092 or placebo	No data/intravenous (IV) infusion (once every 4 weeks or once every 12 weeks)	Evaluation of the safety and tolerability of BIIB092 in participants Evaluation of the efficacy of multiple doses of BIIB092 in slowing cognitive and functional impairment in participants Evaluation of the long-term safety and tolerability of BIIB092 in participants	50 to 80 years	With mild cognitive impairment due to AD or with mild AD	[172]
NCT03959553	A multicenter, randomized, double-blind, placebo-controlled, dose finding, parallel-group study	IIa	GV1001	0.56 mg and 1.12 mg/subcutaneously (once weekly for 4 weeks then every 2 weeks through week 24)	Evaluation of the efficacy and safety of GV1001	55 to 85 years	With moderate AD	[173]
NCT03625622	A double-blinded, randomized, placebo-controlled study	II	AR1001	10 mg or 30 mg/orally (once daily for 26 weeks)	Evaluation of the efficacy and safety of AR1001	50 to 80 years	With mild to moderate AD	[174]
NCT03507790	A multi-center, randomized, double-blind, placebo-controlled, parallel group multicenter study	II	CT1812	100 mg or 300 mg/administered once daily for 6 months	Evaluation of the safety of two doses of CT1812 Evaluation of the effect of CT1812 on biomarkers	50 to 85 years	with mild to moderate AD	[175]
NCT03991988	A one-year, double-blind placebo-controlled randomized clinical trial that compares montelukast to placebo	II	Montelukast	10, 20 to 40 mg	Evaluation of the cognitive function, CSF biomarkers and neuroimaging (cerebral perfusion and markers of vascular brain damage)	50 years and older	with mild cognitive impairment and early AD dementia	[176]

## Data Availability

Not applicable.

## Abbreviations

The following abbreviations are used in this manuscript:

AD	Alzheimer’s disease
APP	amyloid precursor protein
Aβ	amyloid-β protein
APOE	gene encoding apolipoprotein E
apoE	apolipoprotein E
CSF	cerebrospinal fluid
ABCA1	ATP-binding cassette transporter
AHP	Ca (2^+^)-dependent hippocampal after hyperpolarization
AβOs	amyloid-β oligomers
NHPs	non-human primates
PKA	protein kinase A
BBB	blood/brain barrier
RBANS	Repeatable Battery for the Assessment of Neuropsychological Status
MMSE	Mini-Mental State Examination
VEGF	Vascular endothelial growth factor
SSRI	selective serotonin reuptake inhibitors
RV	resveratrol
SIRT1	silent information regulator-1
GSK-3β	glycogen synthase kinase-3β
